# A Novel W1999S Mutation and Non-Target Site Resistance Impact on Acetyl-CoA Carboxylase Inhibiting Herbicides to Varying Degrees in a UK *Lolium multiflorum* Population

**DOI:** 10.1371/journal.pone.0058012

**Published:** 2013-02-28

**Authors:** Shiv Shankhar Kaundun, Geraldine C. Bailly, Richard P. Dale, Sarah-Jane Hutchings, Eddie McIndoe

**Affiliations:** Syngenta, Jealott's Hill International Research Centre, Bracknell, United Kingdom; University of Umeå, Sweden

## Abstract

**Background:**

Acetyl-CoA carboxylase (ACCase) inhibiting herbicides are important products for the post-emergence control of grass weed species in small grain cereal crops. However, the appearance of resistance to ACCase herbicides over time has resulted in limited options for effective weed control of key species such as *Lolium* spp. In this study, we have used an integrated biological and molecular biology approach to investigate the mechanism of resistance to ACCase herbicides in a *Lolium multiflorum* Lam. from the UK (UK21).

**Methodology/Principal Findings:**

The study revealed a novel tryptophan to serine mutation at ACCase codon position 1999 impacting on ACCase inhibiting herbicides to varying degrees. The W1999S mutation confers dominant resistance to pinoxaden and partially recessive resistance to cycloxydim and sethoxydim. On the other hand, plants containing the W1999S mutation were sensitive to clethodim and tepraloxydim. Additionally population UK21 is characterised by other resistance mechanisms, very likely non non-target site based, affecting several aryloxyphenoxyproprionate (FOP) herbicides but not the practical field rate of pinoxaden. The positive identification of wild type tryptophan and mutant serine alleles at ACCase position 1999 could be readily achieved with an original DNA based derived cleaved amplified polymorphic sequence (dCAPS) assay that uses the same PCR product but two different enzymes for positively identifying the wild type tryptophan and mutant serine alleles identified here.

**Conclusion/Significance:**

This paper highlights intrinsic differences between ACCase inhibiting herbicides that could be exploited for controlling ryegrass populations such as UK21 characterised by compound-specific target site and non-target site resistance.

## Introduction

Acetyl-CoA carboxylase (ACCase) is an ubiquitous enzyme that catalyses the carboxylation of acetyl-CoA into malonyl-CoA [1]. Plants have two different forms of ACCase located in the cytoplasm and chloroplast [2]. The two different isoforms share around 60% similarity at the amino acid level and perform different functions [3]. Plastid ACCase is a key enzyme in *de novo* fatty acid synthesis whilst cytosolic ACCase provides malonyl-CoA for the elongation of long chain fatty acid and synthesis of secondary plant metabolites such as flavonoids and suberins [4]. The cytosolic ACCase of all plants are homomeric with the four subdomains biotin carboxyl carrier protein (BCCP), biotin carboxylase (BC) and carboxyl transferase α and β, all located on a single polypeptide [5]. In contrast, the structure of plastidic ACCase varies depending on plant groups. Chloroplastic ACCase is homomeric in the Poaceae, and heteromeric in most other plants with the four subunits encoded by four different genes co-ordinately expressed to form a functional enzyme [6].

This difference between grasses and broadleaved weeds has been exploited by the agro-industry with the development of three different classes of ACCase inhibiting herbicides including the aryloxyphenoxypropionates (FOPs), cyclohexanediones (DIMs) and phenylpyraxolin [7], [8]. These herbicides are generally active on the chloroplast ACCase of most grass weeds but show very little to no efficacy on the cytoplasmic isoform [9]. Over the last 30 years, 18 ACCase herbicides have been marketed providing excellent control of grass weeds.

Given their cost-effectiveness and convenience for managing grass weeds post-emergence, ACCase herbicides were quickly adopted and often used year after year in monocotyledonous and the subsequent rotational dicotyledonous crops. The repeated use of ACCase herbicides has resulted in resistance evolution in a growing number of grass weeds. To date, 42 different weeds species have been reported to resist at least one ACCase herbicide [10]. These include rigid ryegrass (*Lolium rigidum*), the first species to have evolved resistance to an ACCase herbicide following six years of diclofop-methyl use in a wheat field in Australia [11].

Two types of mechanisms account for resistance to ACCase herbicides, namely, an insensitive target and/or non-target site based resistance [12]. Target site resistance results from changes in amino acid residues that are critical for the optimal binding of the herbicide. Mutations at codon positions 1781, 1999, 2027, 2041, 2078, 2088 and 2096 (*Alopecurus myosuroides* equivalent) are documented to confer resistance to ACCase herbicides [3], [13]. Genotype to phenotype correlation experiments show that specific amino acid changes at the seven codon positions can have very diverse effects between and within ACCase chemical classes [14], [15], [16], [17]. The levels of resistance depend on the herbicides, use rates, weed species and number of resistant alleles in individual plants [18]. Non-target site based resistance to ACCase herbicide appears to be widespread but less well understood than target site resistance [19], [20]. It consists mainly of enhanced degradation of ACCase herbicides into non-toxic metabolites [21]. Metabolic resistance is unpredictable and can impact on herbicides with similar or even different herbicide modes of action [22]. Genetic studies have shown that metabolic resistance is complex with several loci acting additively to confer resistance to ACCase herbicides [23], [24], [25].

While resistance to ACCase herbicides has evolved in all major cropping systems worldwide, a significant proportion of grass weed populations are still sensitive to this class of herbicides [10]. In order to design weed management strategies that will delay the onset and sometimes overcome resistance, it is important to better understand the mechanisms by which weeds evolve resistance to ACCase herbicides. Here we have employed a holistic biological and molecular approach to investigate the mechanisms of resistance to a wide range of ACCase inhibiting herbicides in a *Lolium multiflorum* population (UK21) from the UK. We have subsequently developed a robust DNA marker to quickly and cost-effectively identify the novel target site resistance mutation detected in this study.

## Materials and Methods

### Materials

Seeds from a suspected pinoxaden resistant *Lolium multiflorum* population UK21 were collected from a wheat field in Chislet, Canterbury, UK in 2006. A standard sensitive population (STD1) was sourced from Herbiseed (Twyford, UK) for comparison in all biological and molecular experiments.

“No specific permissions were required for the location where the ryegrass seeds were collected. This study did not involve any endangered or protected species”.

### Methods

#### Pinoxaden resistance confirmation test

The suspected resistant and sensitive populations were sown in 5 inch pots in soil composed of peat and compost in 1∶1 ratio. Around 50 seeds were sown per population per pot. The pots were watered daily, fertilized as required and placed in controlled glasshouse conditions set at 24°C/16 hr day, 18°C night, 65% relative humidity, and a photon flux density of approximately 250 μmol quanta m^−2^ s^−1^. When the plants had reached the two leaf stage, they were sprayed with a single recommended rate of pinoxaden at 45 g ai ha^−1^ in a spray cabinet mounted with a single mobile Teejet flat fan nozzle (11002VS) calibrated to deliver 200 L ha^−1^ at 200 kPa. Three replicate pots were used per population and the percentage of herbicide damage compared with untreated pots was assessed visually three weeks after treatment.

#### Selection of pinoxaden sensitive and resistant plants

Around 100 seeds from each of UK21 and STD1 were sown in two rectangular trays and grown as described above. At the one leaf stage 48 seedlings from each of the two populations were individually transplanted in 3 inch pots and maintained under the same glasshouse conditions until they reached the two leaf stage. A 1 cm leaf fragment was collected from each plant for ACCase gene sequencing analysis prior to spraying with pinoxaden at the recommended field rate of 45 g ai ha^−1^. Three weeks after treatment the plants were assessed for survival.

#### ACCase gene sequencing from selected sensitive and pinoxaden resistant plants

To determine whether a mutated target was responsible for pinoxaden resistance, the ACCase carboxyltranferase domain was sequenced for 8 UK21 plants that were killed and 8 UK21 plants that survived the pinoxaden treatment. Eight other plants from the standard sensitive population STD1 were analysed in a similar way for comparison. The RT-PCR procedure for generating and sequencing the ACCase gene fragment encompassing the carboxyl transferase domain was the same as described in Kaundun (2010) [26].

#### Development of a dCAPS assay for large scale genotyping of UK21 plants

A derived cleaved polymorphic sequence assay (dCAPS) [27] was developed for the positive identification of the wild type tryptophan and mutant serine alleles in *Lolium* spp. plants. The primers and corresponding restriction enzymes were chosen using the dCAPS freeware [28]. The assay consisted of a single PCR fragment and two different restriction enzymes, namely *Xcm*I for positively detecting the wild type 1999 tryptophan allele and *Mnl*I for identifying the mutant serine allele in two separate reactions. Changes in any one of the three bases of the wild 1999 codon would disrupt the *Xcm*I restriction site and result in an undigested band on the agarose gel. On the other hand, restriction with *Mnl*I would indicate presence of a mutant serine allele or nucleotides TC on the first and second bases of the 1999 codon. The recognition of the wild type tryptophan and mutant serine alleles was rendered possible by the introduction of three forced mutations (underlined bases) in the forward dCAPS primer (5′ cccatgagcggtccgttc**t**tcgtccagggcaagcc 3′). The forced mutations at positions N-13 (guanine to cytosine) and N-11 (thymine to adenine) with respect to the second variable base of the 1999 codon create the *Xcm*I recognition site CCA(N)9TGG for plants with the wild type tryptophan allele. The forced nucleotide change at position N-3 with respect to the causative resistance SNP introduces a recognition site for *Mnl*I (CCTC) for plants carrying the mutant 1999 serine allele. The nucleotide sequence for the reverse primer was: 5′ aatccttcaaaaaggtctctttgcccaccagagaagc**t**tctccagttagcaaggatgaaca**a**agg3′ for both dCAPS reactions. Noteworthy is three other forced mutations in all (in bold) were included on the forward (N-18) and reverse (N+90; N+66) primers with respect to the mutated second base of the 1999 codon to eliminate three non-specific restriction sites for *Mnl*1. If left unchanged, shorter and ambiguous digests which would be difficult to assess on a 2% agarose gel would be generated. The 8 plants each of sensitive UK21, resistant UK21 and STD1 groups previously sequenced for the entire ACCase CT domain were used to develop the *Xcm*I and *Mnl*I based assays. The three step dCAPS assays were carried out as follows: for each plant a 1 cm leaf segment was ground on a Spex Certiprep (Metuchen, NJ, USA) 2000 model Genogrinder; DNA from the ground material was subsequently extracted on a Beckman Coulter Biomek FX robot (High Wycombe, Buckinghamshire, UK) using a Wizard Magnetic 96 DNA Plant System kit (Promega, Madison, WI, USA). PCRs were carried out using PuReTaq Ready-To-Go PCR beads (Amersham Biosciences, Bucks, UK) in a total volume of 25 µL containing 0.8 µM of each primer and from 10–50 ng genomic DNA. PCRs were performed on an Eppendorf Master Cycle Gradient Thermocycler Model 96 programmed for an initial denaturation step of 94°C of 2 min followed by 40 cycles of 30 s at 94°C, 30 s at 64°C and 1 min at 72°C. A final extension step for 10 min at 72°C was also included. For the positive identification of the wild type TGG codon characteristic of the tryptophan residue, 8 µl of the PCR product was then digested with 1 µL (5 units) of *Xcm*I at 37°C for 2 hours in a 40 µL reaction. Ten µL of the PCR product treated with *Xcm*I was then migrated on a 1xTBE 2% agarose gel containing 0.5 µg mL−1 ethidium bromide. The same procedure and conditions were used for the positive identification of the mutant serine 1999 allele except that *Xcm*I was replaced by the enzyme *Mnl*I.

#### Correlation between plant genotypes and phenotypes at single recommended rates of eight ACCase herbicides

To investigate the influence of the tryptophan to serine mutation at ACCase codon position 1999 and potential non-target site resistance mechanisms on eight other ACCase herbicides, 384 UK21 individual plants were grown to the two leaf stage as described above and genotyped using the *Xcm*I based dCAPS assay. Forty eight random UK21 plants each were subsequently sprayed with the recommended field rates of diclofop-methyl (1000 g ai/ha), clodinafop-propargyl (60 g ai/ha), fluazifop-P-butyl (200 g ai/ha), haloxyfop-P-methyl (100 g ai/ha), sethoxydim (200 g ai/ha), cycloxydim (200 g ai/ha), tepraloxydim (100 g ai/ha) and clethodim (120 g ai/ha). For comparison, 48 plants each from the standard sensitive population were also sprayed with the same eight herbicides. Survival to the herbicides was assessed 21 days after treatment. Plant survival data from the co-segregation experiment were arranged as a 2×2 contingency table for each genotype comparison and analysed using Fisher's Exact test (Table 1). Formal analyses were only carried out for haloxyfop-P-methyl, sethoxydim, cycloxydim and pinoxaden since all other compounds produced either 0% or 100% survival across all three WW1999 (homozygous wild type with two copies of the tryptophan allele), WS1999 (heterozygous mutant type with one copy each of the tryptophan and serine alleles) and SS1999 (homozygous mutant type with two copies of the serine allele) genotypes. A p-value of less than 0.05 indicates a statistically significant result at the 5% probability level and provides evidence that the true levels of survivorship in the two treatments in question are genuinely different.

**Table 1:** Correlation between plant phenotypes and genotypes at ACCase codon position 1999.

			No. of survivors/Total		
Compound	Rate (g/ha)	Comparison	Group1	Group2	P-value (2-sided)
Pinoxaden	45	WW v WS	0/12	22/22	<**0.001**
		WW v SS	0/12	14/14	<**0.001**
		WS v SS	22/22	14/14	1
Haloxyfop-P-methyl	100	WW v WS	4/9	25/31	***0.047***
		WW v SS	4/9	8/8	***0.029***
		WS v SS	25/31	8/8	0.313
Sethoxydim	200	WW v WS	0/10	6/26	0.1567
		WW v SS	0/10	7/12	0.**0053**
		WS v SS	6/26	7/12	0.0636
Cycloxydim	200	WW v WS	0/13	4/21	0.144
		WW v SS	0/13	6/14	***0.016***
		WS v SS	4/21	6/14	0.251

A significant difference at the 5% probability level is shown in bold.

Additionally given the complete resistance observed with the three FOP herbicides, 8 plants that have survived clodinafop-propargyl and that have shown a wild type allele at codon position 1999 with the dCAPS assay was sequenced with the RT-PCR procedure described above to determine whether any FOP specific target site mutations were present in population UK21.

#### Whole plant dose-response experiments using pre-determined 1999 ACCase genotypes

In order to determine the level of resistance conferred by target site and suspected non-target site resistance identified in UK21, whole plant dose-response experiments were conducted using known plant genotypes at ACCase codon position 1999. The genotypes were characterised with the dCAPS and sequencing assays described previously. Four mother plants for each of the WW1999, WS1999 and SS1999 genotypes were selected from population UK21. Four mother plants from the standard sensitive population STD1 (WW1999) were included for comparison. This amounted to four plant groups with four biological replicates each. Individual plants were subsequently tiller propagated over a period of one year under the same glasshouse conditions as described previously. Tillering was promoted by regularly fertilizing and trimming the plants to avoid flowering. One genetically identical tiller from each of the 16 mother plants were sprayed with several rates of diclofop-methyl (Hoelon 3EC, 347 gai.L^−1^, Bayer Crop Science), sethoxydim (Grasidim, 213 gai.L^−1^, BASF Corporation), haloxyfop-P-methyl (Gallant 535, 108 gai.L^−1^, Dow AgroSciences), tepraloxydim (Aramo 50, 50 gai.L^−1^, BASF Corporation) and pinoxaden (Axial, 100 gai.L^−1^, Syngenta) (Table 2). Four tillers per mother plants were used as untreated control. The dose-responses were repeated two times in time (technical replicates). The pots were re-arranged in a randomized complete block design after herbicide application (blocking per mother plant). Twenty-one days after treatment (DAT) the tillers were cut above ground level, put into paper envelopes and dried in an oven at 70°C for at least three days. The dry mass for each individual was recorded to the nearest mg.

**Table 2:** Summary of herbicide rates used in the whole-plant dose-response experiments.

Genotypes	Pinoxaden	Diclofop-methyl	Haloxyfop-P-methyl	Sethoxydim	Tepraloxydim
STD1-WW1999	1; 2; 4; 7; 13; 23; **45** and 95	10; 25; 56; 93; 155; 258; 431; 719; **1,000** and 2,233	0.1; 0.25; 0.63; 2.5; 12; 25; 64; **100** and 400	5; 13; 25; 37; 60; 95; 152; **200**; 314 and 400	0.1; 0.24; 0.59; 1; 5; 8; 20; 50; **100** and 300
UK21-WW1999	1; 2; 4; 7; 13; 23; **45** and 95	**1,000**; 1,351; 1,825; 2,233; 3,332; 4,502 and 6,082	5; 8; 12; 25; 39; 64; **100**; 151; 228 and 400	5; 13; 25; 37; 60; 95; 152; **200**; 314 and 400	0.1; 0.24; 0.59; 1; 5; 8; 20; 50; **100** and 300
UK21-WS1999	23; **45**; 95; 145; 279; 472; 800 and 1,000	**1,000**; 1,351; 1,825; 2,233; 3,332; 4,502; 6,082; 8,217; 11,102 and 15,000	25; 39; 64; **100;** 228; 400; 608; 1,035; 1,762 and 3,000	13; 25; 37; 60; 95; 152; **200**; 314; 604 and 1,000	5; 8; 12; 20; 31; 50; 77; **100**; 190 and 300
UK21-SS1999	**45**; 95; 145; 208; 346; 577; 1,000 and 1,600	**1,000**; 1,351; 1,825; 2,233; 3,332; 4,502; 6,082; 8,217; 11,102 and 15,000	25; 39; 64; **100**; 228; 400; 608; 1,035; 1,762 and 3,000	13; 25; 37; 60; 95; 152; **200**; 314; 604 and 1,000	5; 8; 12; 20; 31; 50; 77; **100**; 190 and 300

The rates are indicated in g ai.ha^−1^. The rates in bold represent the recommended field rate for ryegrass control. All herbicides were formulated in dH_2_O using their recommended adjuvant: 0.5% v/v Actipron for sethoxydim and diclofop-methyl; 0.375% v/v Output for haloxyfop-P-methyl and tepraloxydim and 0.5% v/v Adigor for pinoxaden.

Since the mother plants represent the independent biological replicates in the experiment, the dry weight measurements were averaged across technical replicates (time) separately for each compound, rate and mother plant and divided by the average dry weight of the untreated tillers from the corresponding mother plant. GR_50_ estimates were derived from the resulting percent of untreated values separately for each mother plant by a non-linear least-squares regression model described by the equation:
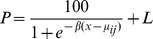
where x denotes log(Rate); µ_ij_ denotes the logGR_50_ for mother plant i of genotype j; β denotes the common slope and L denotes the common lower asymptote fitted to all regression lines. The regression line for each mother plant has 3 parameters (a slope, logGR_50_ and lower asymptote) resulting in a model with 18 parameters in total: 1 slope, 1 lower asymptote and 16 logGR_50_ values.

The estimated resistance factor (Rf) between a pair of genotypes is calculated as the ratio of their average GR_50_ values across mother plants. Confidence intervals around the estimated resistance factors were calculated using the error term derived from an analysis of variance carried out on the logGR_50_ values. All statistical analyses were carried out using SAS software, version 9.2.

## Results

### Initial whole plant pot test for confirming resistance to pinoxaden

Pinoxaden treatment at the recommended field rate provided 35±2.9% control (visual biomass reduction) of the suspected resistant population UK21. The individuals that have survived varied from being very stunted to being as healthy as untreated plants. As expected all the plants from the standard sensitive population STD1 were killed. Though pinoxaden is a cereal selective and metabolisable herbicide, to date most documented cases of product failures were due to a target site mutation in *Lolium* spp. [16], [17], [19], [29]. Therefore, a mutated and insensitive target rather that non-target site resistance was suspected to being the most likely mechanism of resistance to pinoxaden in population UK21.

### Segregation of the UK21 population into pinoxaden sensitive and resistant individuals and ACCase gene analysis

**Figure 1:**
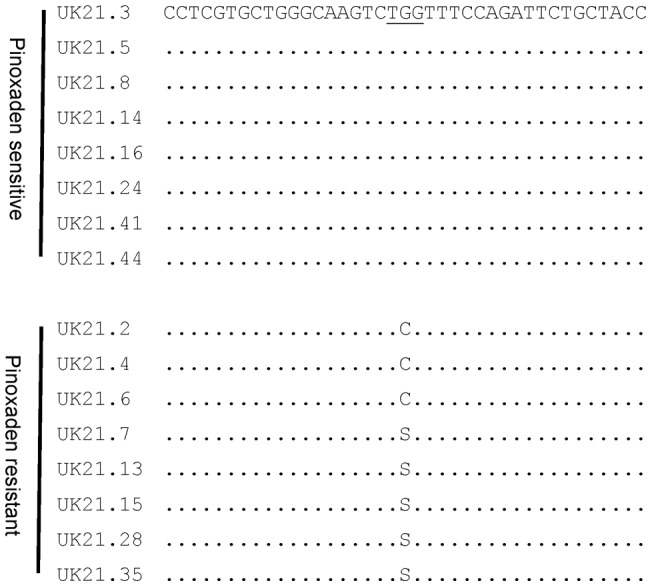
ACCase gene alignment around the critical nucleotide position 5996 (second base of the 1999 codon) among eight plants sensitive and resistant to pinoxaden. All sensitive plants contained two copies of guanine (gg) whilst resistant plant had one (cg  = s) or two copies of cytosine (cc) at this position.

Out of 48 individual plants tested, 36 survived the pinoxaden treatment. Polymerase chain reaction from eight random plants each from the sensitive and resistant UK21 subpopulations and the standard sensitive population STD1 generated a 2272 gene fragment in all cases. Analysis of the nucleotide sequences from the 24 plants showed 95% homology with published data, thus confirming the identity of the ACCase carboxyl transferase domain amplified here. Sequence comparison identified 15 nucleotides changes in all among those 24 plants. Nine of the nucleotide substitutions were synonymous and thus did not amount to differences in amino acid sequences between and within the two ryegrass populations. Five single nucleotide polymorphisms were non-synonymous at amino acid positions L1701, E1874 E1946, R1995 and T2054 but these were evenly distributed among the three different plant groups. In contrast, a tryptophan to serine mutation at amino acid position 1999 (W1999S) was only present in the eight UK21 resistant plants but absent in the sensitive UK21 as well as the standard sensitive STD1 samples (Figure 1). Out of the eight resistant plants, five were heterozygous WS1999 and three were homozygous for the serine 1999 mutation.

### dCAPS assay for the rapid and cost effective identification of the W1999S mutation

**Figure 2:**
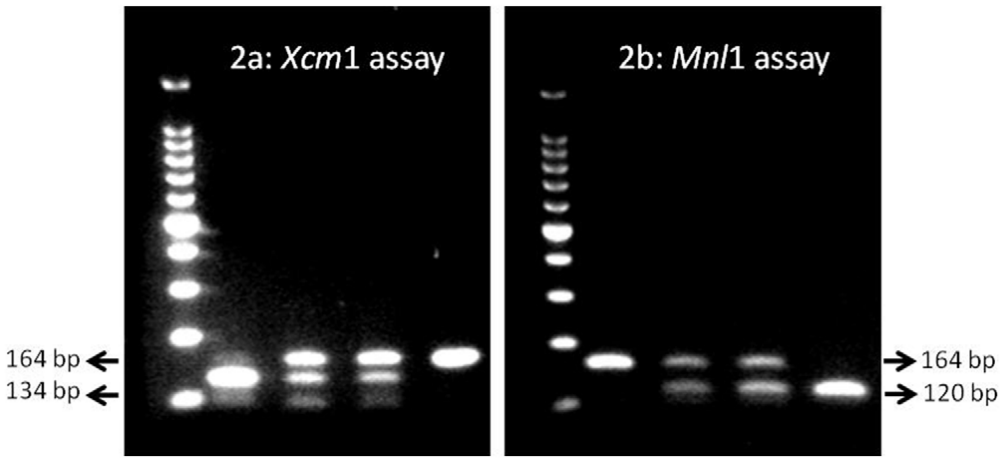
dCAPS procedures for the detection of the wild type tryptophan and mutant serine amino acid residues at ACCase codon position 1999: (a) *Xcm*I restricted (134 bp) and unrestricted (164 bp) fragments correspond to sensitive W1999 and resistant S1999 ACCase alleles respectively. (b) *Mnl*I restricted fragment is indicative of the mutant serine allele (120 bp) while the undigested band (164 bp) corresponds to the wild type tryptophan allele. Heterozygous plants display one each of the restricted and unrestricted PCR fragment in both assays.

A dCAPS assay was developed for large scale genotyping of single UK21 plants in view of confirming the association between the 1999 mutation and pinoxaden resistance. Digestion with two different enzymes allowed the positive identification of the wild type tryptophan or mutant serine alleles. Typical dCAPS profiles as resolved on 2% agarose gel electrophoresis are provided in figures 2a and 2b. PCR generated a 164 bp fragment for all plants. When digested with the enzyme *Xcm*1, wild type homozygous plants showed a single restricted band of 134 bp (and 30 bp fragment not visible of 2% agarose gel) while plants that are homozygous for the serine 1999 allele displayed the undigested PCR fragment of 164 bp. As expected, heterozygous plants showed a copy each of the 164 and 134 bp fragments. The inverse restriction profiles were obtained with the enzyme *Mnl*I with plants containing the wild type tryptophan allele being unrestricted whilst individuals with the mutant serine allele showed a shorter digested band of 120 bp. It is noteworthy that the difference in size of restricted PCR fragments between *Xcm*I and *Mnl*I treatments is due to the fact that the second enzyme generally cuts 6/7 bp downstream of the CCTC recognition site while the first enzyme cleaves 4/5 base pairs upstream of the TGG 1999 codon. The dCAPS and sequencing results for the 16 UK21 and 8 STD1 plants were totally correlated demonstrating the accuracy of the dCAPS assay. Genotyping of the 32 remaining UK21 plants (that were phenotyped with pinoxaden at a single recommended rate) with the *Xcm*I based dCAPS assay confirmed the complete association between the presence of the mutated serine 1999 ACCase allele and resistance to pinoxaden (Table 1).

### Cross resistance patterns to a range of ACCase herbicides

Analysis of 384 UK21 individuals in all with the *Xcm*I dCAPS marker revealed genotypic frequencies of 0.199, 0.598 and 0.203 respectively for wild type WW1999, heterozygous mutant WS1999 and homozygous mutant SS1999 plants in population UK21. These were randomly separated into eight lots of 48 plants and tested with eight different herbicides alongside the standard sensitive population. As expected, plants from the latter population were controlled with the eight ACCase herbicides. All UK21 plants survived the clodinafop-propargyl, diclofop-methyl and fluazifop-P-butyl treatments irrespective of their genotypes at ACCase codon position 1999. Sequencing of eight plants that have survived clodinafop-propargyl and containing a wild type allele at codon position 1999 did not reveal any other specific mutations compared to the sensitive population suggesting that additional resistance to the FOP herbicides in UK21 is very probably non-target site based. Conversely all the wild type and mutant UK21 plants were killed at the recommended field rates of tepraloxydim and clethodim. Intermediate responses were observed with haloxyfop-P-methyl, sethoxydim and cycloxydim (Table 1). Haloxyfop-P-methyl behaved in a very similar manner to pinoxaden, given that all the homozygous mutant SS1999 plants and a very large proportion of heterozygous WS1999 plants survived this FOP treatment. Additionally, around half of the wild type UK21-WW1999 plants also survived the haloxyfop-P-methyl treatment. Formal statistical analysis of the data revealed a significant difference between the heterozygous mutant UK21-WS1999 and the wild type UK21-WW1999 subpopulations implying that the W1999S mutation confers dominant resistance to haloxyfop-P-methyl in UK21. Sethoxydim and cycloxydim killed all the wild type UK21-WW1999 plants. In contrast to pinoxaden, only around half of the homozygous mutant UK21-SS1999 plants and an even lower proportion of heterozygous UK21-WS1999 plants survived sethoxydim (23%) and cycloxydim (19%) treatments. No convincing evidence of a difference in survivorship between heterozygous UK21-WS1999 and homozygous wild type UK21-WW1999 plants was identified with the Fisher exact test for these two DIM herbicides. On the other hand, there was evidence of a difference between homozygous wild type UK21-WW1999 and mutant UK21-SS1999 individuals suggesting that resistance could be classified as partially recessive to sethoxydim and cycloxydim.

### Level of resistance conferred by the W1999S target site and non-target site resistance

**Figure 3:**
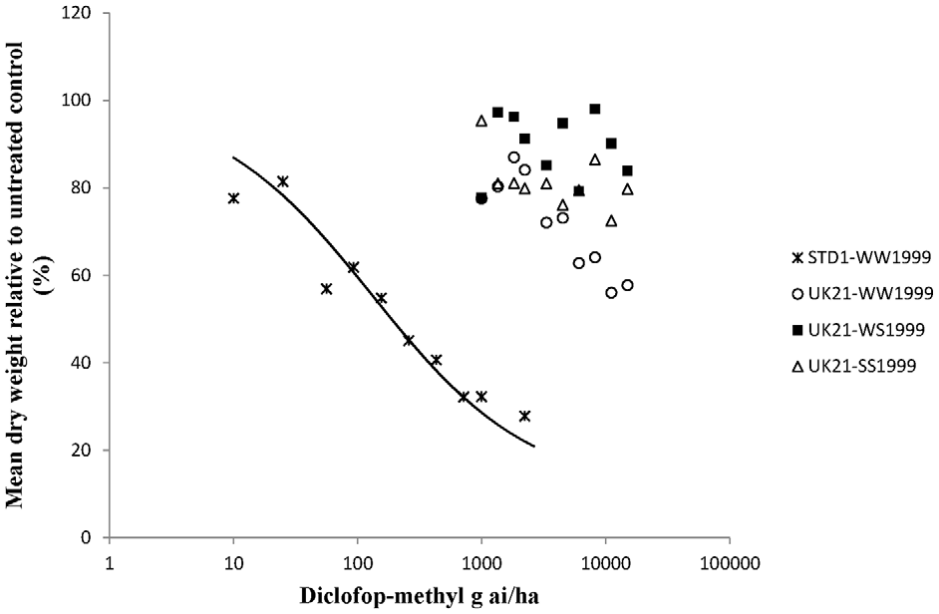
Diclofop-methyl dose response tests on four plant groups: homozygous wild type WW1999, heterozygous WS1999 and homozygous mutant SS1999 from the mixed resistant population UK21 and standard sensitive STD1 plants (WW1999) for comparison. Observed values represent dry weight relative to untreated (%) averaged across mother plants. The curves are constructed based on the average GR50 across biological and technical replicates.

**Figure 4:**
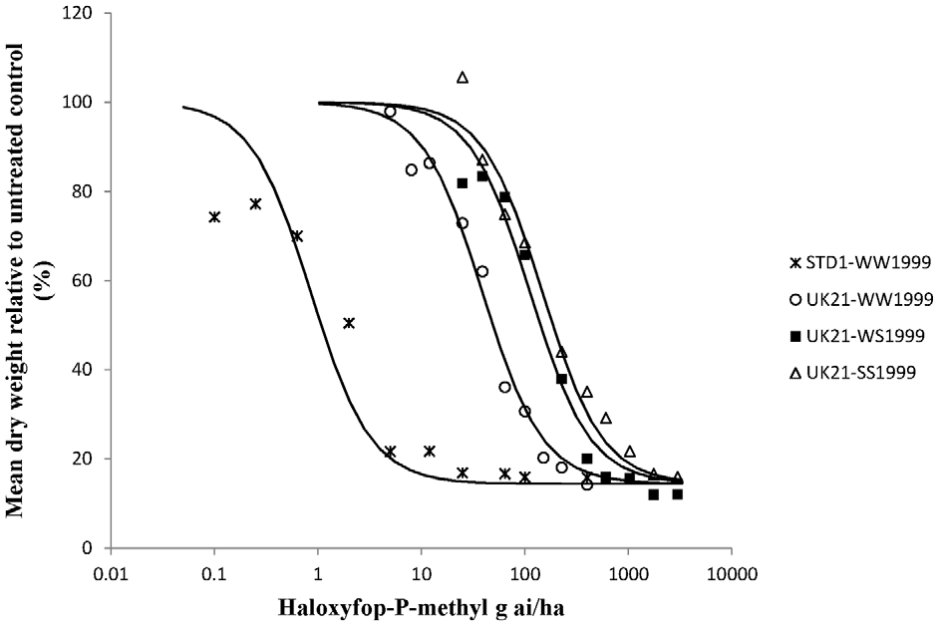
Haloxyfop-P-methyl dose response tests on four plant groups: homozygous wild type WW1999, heterozygous WS1999 and homozygous mutant SS1999 from the mixed resistant population UK21 and standard sensitive STD1 plants (WW1999) for comparison. Observed values represent dry weight relative to untreated (%) averaged across biological and technical replicates.

**Figure 5:**
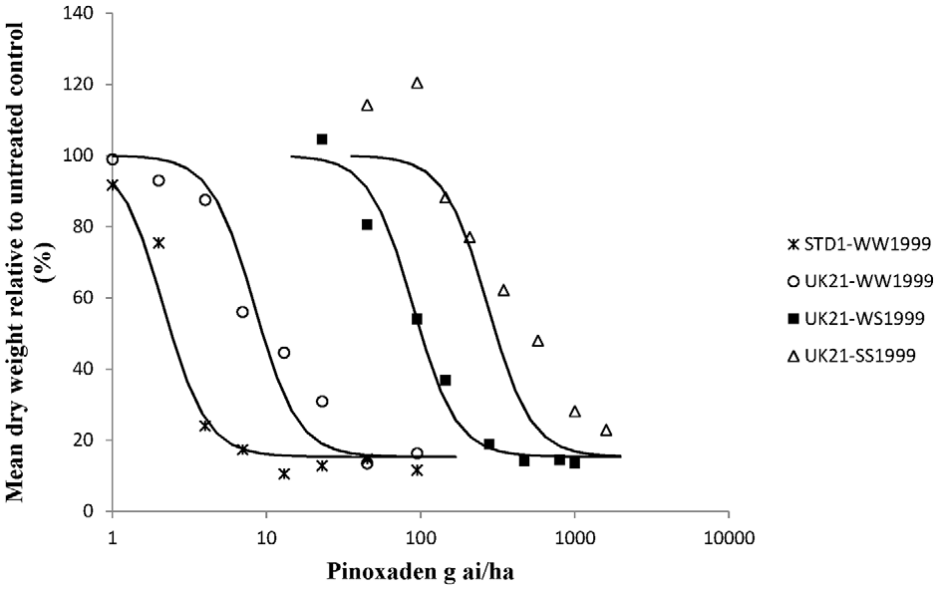
Pinoxaden dose response tests on four plant groups: homozygous wild type WW1999, heterozygous WS1999 and homozygous mutant SS1999 from the mixed resistant population UK21 and standard sensitive STD1 plants (WW1999) for comparison. Observed values represent dry weight relative to untreated (%) averaged across biological and technical replicates.

**Figure 6:**
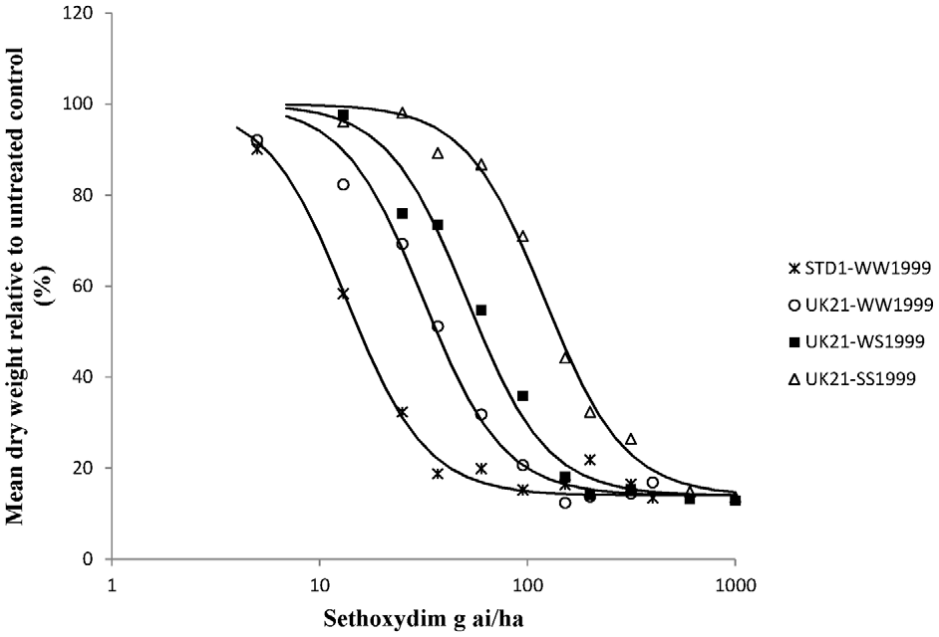
Sethoxydim dose response tests on four plant groups: homozygous wild type WW1999, heterozygous WS1999 and homozygous mutant SS1999 from the mixed resistant population UK21 and standard sensitive STD1 plants (WW1999) for comparison. Observed values represent dry weight relative to untreated (%) averaged across mother plants. The curves are constructed based on the average GR50 across biological and technical replicates.

**Figure 7:**
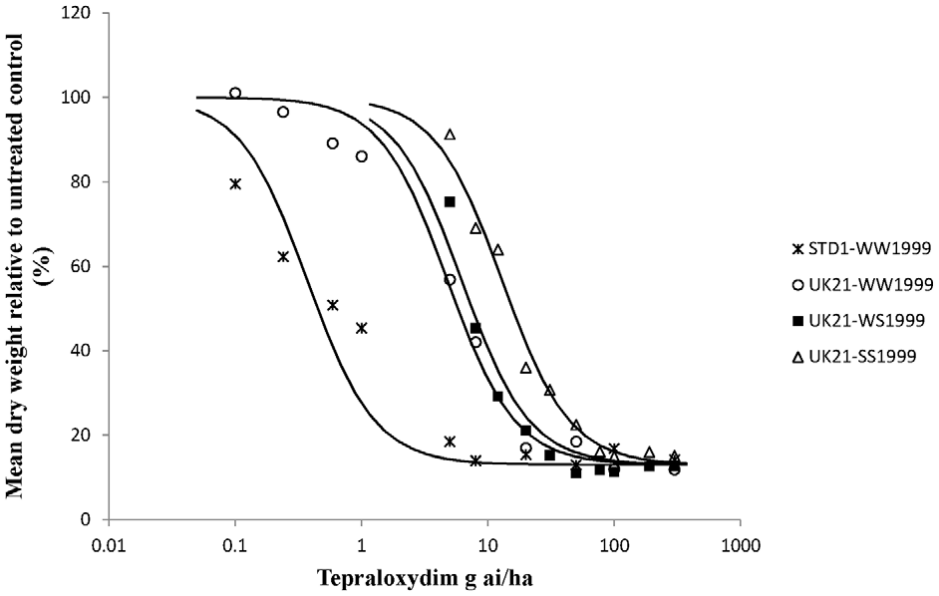
Tepraloxydim dose response tests on four plant groups: homozygous wild type WW1999, heterozygous WS1999 and homozygous mutant SS1999 from the mixed resistant population UK21 and standard sensitive STD1 plants (WW1999) for comparison. Observed values represent dry weight relative to untreated (%) averaged across mother plants. The curves are constructed based on the average GR50 across biological and technical replicates.

Whole plant dose response tests on characterised UK21 genotypes were carried out to determine the precise levels of resistance conferred by target site and non-target site resistance to two FOPs, two DIMs and pinoxaden in UK21 (Tables 3 and 4). Dose response curves were obtained for all plant groups and herbicides except for diclofop-methyl on all three different UK21 genotypes (Figure 3). At the highest rate tested (15 kg ai/ha) all the plants survived the diclofop-methyl treatment. On average, the heterozygous and homozygous mutant plants (<20% biomass reduction) were less controlled than the homozygous wild type plants (42% biomass reduction) implying that the target site resistance at position 1999 did contribute to higher levels of resistance to diclofop-methyl in UK21. Compared to diclofop-methyl, lower levels of resistance were observed for haloxyfop-p-methyl with respect to the W1999S target site resistance and non-target site resistance contained in UK21 (Figure 4). One or two copies of the mutant serine allele amounted to a resistance factor (Rf) of around 3 in both cases. However this was sufficient to cause survivorship to the FOP herbicide at the recommended field rate. A higher level of resistance was identified between the wild type UK21-WW1999 and STD1-WW1999 plants. Resistance to pinoxaden was confirmed with the W1999S mutation resulting in resistance factors of 11 and 33 for one and two mutant serine alleles respectively (Figure 5). The shift between the wild type UK21-WW1999 and STD1-WW1999 plants was small (Rf of 3) with average GR50 values of 2.1 g ai/ha and 8.3 g ai/ha only, thus confirming the non-significance of non-target site resistance at the recommended field rate of pinoxaden. The W1999S mutation had a lesser effect on sethoxydim compared to pinoxaden reflecting the partially recessive and dominant nature of this mutation on the DIM and DEN herbicide respectively (Figure 6). And finally, the dose response assay ascertained the minimal impact of the W1999S mutation on tepraloxydim with almost overlapping curves for UK21-WW1999 and UK21-WS1999 (Figure 7). GR50 values for any one wild and mutant UK21 genotypes were well below the recommended field rate of the herbicide (Table 3).

**Table 3:** Estimated GR50 values for five ACCase herbicides and different 1999 ACCase genotypes.

	STD1-WW1999	UK21-WW1999	UK21-WS1999	UK21-SS1999
Pinoxaden	2.1 (0.7–6.7)	8.4 (2.7–26.1)	89.2 (28.6–278.5)	272.9 (87.4–851.9)
Diclofop-methyl	132.1 (1.5–11290.8)	>6082	>15000	>15000
Haloxyfop-P-methyl	0.9 (0.3–2.3)	39.1 (14.4–105.9)	112.7 (41.6–305.0)	148.7 (54.9–402.5)
Sethoxydim	13.5 (9.2–19.7)	31.6 (21.7–46.1)	51.2 (35.1–74.6)	121.4 (83.3–177.0)
Tepraloxydim	0.4 (0.2–0.7)	4.8 (2.4–9.5)	6.2 (3.2–12.3)	13.0 (6.6–25.7)

95% confidence limits in brackets.

**Table 4:** Estimated resistance factors for five ACCase herbicides for different 1999 ACCase plant groups.

Genotypes	Pinoxaden	Diclofop-methyl	Haloxyfop-P-methyl	Sethoxydim	Tepraloxydim
UK21-WW1999 v STD1-WW1999	3.9 (1.3–12.2)	>46.0	45.5 (16.8–123.2)	2.3 (1.6–3.4)	12.9 (6.5–25.5)
UK21-WS1999 v STD1-WW1999	41.7 (13.3–130.1)	>113.6	131.2 (48.5–355.1)	3.8 (2.6–5.5)	16.7 (8.4–33)
UK21-SS1999 v STD1-WW1999	127.4 (40.8–397.9)	>113.6	173.1 (63.9–468.6)	9.0 (6.2–13.1)	34.8 (17.6–68.8)
UK21-WS1999 v UK21-WW1999	10.7 (3.4–33.3)	-	2.9 (1.1–7.8)	1.6 (1.1–2.4)	1.3 (0.7–2.6)
UK21-SS1999 v UK21-WW1999	32.6 (10.4–101.8)	-	3.8 (1.4–10.3)	3.8 (2.6–5.6)	2.7 (1.4–5.3)
UK21-SS1999 v UK21-WS1999	3.1 (1.0–9.6)	-	1.3 (0.5–3.6)	2.4 (1.6–3.5)	2.1 (1.1–4.1)

UK21- WW1999, UK21-WS1999 andUK21- SS1999 originates from the same population and thus shares the same genetic background except for their amino acids at ACCase position 1999.

STD1 is the standard sensitive population used for comparison.

Resistance factors could not be estimated for diclofop-methyl when no dose responses were observed for both genotypes being compared.

95% confidence limits in brackets.

## Discussion

### Integrated biological and molecular approach for revealing multiple herbicide resistance mechanisms

A growing number of *Lolium* spp. populations worldwide are evolving resistance to the few remaining post-emergence herbicides used to control this species. Additionally the development and registration of new herbicides has become increasingly problematic due to the difficulty of finding new modes of action and stricter regulatory hurdles. Thus, understanding the basis of resistance is imperative for a better stewardship of very effective herbicides such as inhibitors of acetyl-CoA carboxylase. This study reveals a novel target site resistance mutation and other mechanisms, likely non-target site based to several ACCase herbicides in population UK21. Additional underlying non-target site resistance has been inferred in many other *Lolium* spp. [19] *Alopecurus* spp.[30], *Avena* spp. [31] populations that have evolved resistance to ACCase inhibiting herbicides. An exception to this rule is found in a UK ryegrass population characterised by the C2088R ACCase resistance mutation only [32]. The presence of multiple resistance mechanisms often acting additively is expected in a highly polymorphic, outbreeding species such as *Lolium multiflorum*, especially when pressured with the same herbicide mode of action year after year [22]. However, in most previously published studies the contributions of target site and non-target site resistance were not properly evaluated due to heterogeneous resistant weed populations being compared with a standard sensitive population having a very different genetic background. Our approach of creating wild type, heterozygous and homozygous 1999 mutant ACCase sub-populations from the same parental population UK21 and comparison with a standard sensitive population has allowed for a better estimation of the level of resistance conferred by target site and non-target site resistance.

### Differential impact of the W1999S mutation on ACCase herbicides

The novel target site resistance mutation identified in UK21 consists of a change from a tryptophan 1999 to serine (W1999S) amino acid residue in the carboxyltransferase domain of the ACCase enzyme. At ACCase codon position 1999 two other mutations have been identified in grass weeds that have evolved resistance to ACCase herbicides. These include a tryptophan to cysteine change in *Avena sterilis* [14], *Avena fatua* [31] and *Lolium multiflorum* [33], and a tryptophan to leucine change (W1999L) in *Alopecurus myosuroides* [34] and *Lolium multiflorum* [16] populations. The tryptophan 1999 to cysteine (W1999C) mutation was found to confer resistance to fenoxaprop-P-ethyl but not to two other FOP and DIM herbicides in the *Avena sterilis* population [14]. The role of the W1999C mutation on the five different ACCase herbicides was adequately investigated via comparison of plant phenotypes as established with glasshouse herbicide assays and PCR based genotyping at ACCase codon position 1999. As some plants that did not contain the 1999 mutation were also resistant to fenoxaprop-P-ethyl, the importance of the W1999C mutation on ACCase herbicides was further confirmed via yeast-gene replacement assays with strains differing at the 1999 codon position only [14]. On the other hand, the impact of the W1999L remains to be assessed in the blackgrass and ryegrass populations as well as W1999C mutation in the ryegrass and *Avena fatua* populations. In this study the W1999S mutation was found to confer variable levels of resistance to ACCase herbicides within and between the different herbicide subclasses. Clethodim and tepraloxydim controlled all plants containing the serine mutant allele either in homozygous or heterozygous states while the W1999S mutation conferred only partial resistance to the herbicides cycloxydim and sethoxydim. In contrast, the W1999S mutation conferred dominant resistance to haloxyfop-P-methyl and pinoxaden and very probably to the other FOP herbicides tested here.

The difference in the resistance profiles between the *Avena sterilis* [14] and *Lolium multiflorum* populations characterised by the two different W1999C and W1999S mutations is at first sight surprising given that cysteine shares a very similar structure to serine. However, minor differences in amino acid residues such as the isoleucine to leucine change at codon 1781 have previously been shown to have very dramatic effects on the efficacy of ACCase herbicides [3], [18]. The differential impact of the W1999C and W1999S mutations on ACCase inhibiting herbicides could also be due the difference in ploidy levels between these two grass weed species. *Avena sterilis* and *Lolium multiflorum* are hexaploid and diploid species respectively. Thus for *Avena* species, for which all homeologous ACCase genes are expressed [14], the dilution factor is three times more accentuated compared to *Lolium* species when only one of the loci is mutated. While this may have little bearing on plant survivorship for mutations that confer very high levels of resistance to ACCase herbicides such as the I1781L, D2078G, C2088R mutations [13], the number of ACCase gene copies becomes important for amino acid changes that confer lower levels of resistance such as the ones at codon positions 1999. A similar observation was made for the proline 106 mutations in the 3-phosphoshikimate 1-carboxyvinyltransferase glyphosate target. The impact of the P106 mutations, which generally confer low levels of resistance to glyphosate, was greater in *Eleusine indica* characterised by a single *EPSPS* copy compared to *Lolium* species for which the corresponding gene exists as a small family with at least three copies identified. [35]. Therefore the conclusion regarding the impact of the W1999C or W1999L mutations in the ryegrass and blackgrass populations cannot be based on the wild oat study [14] and should be properly evaluated, potentially by using a similar approach to the one employed in this study.

### Occurrence and spread of the W1999S mutation in grass weed species

The mechanism of target site resistance to ACCase herbicides has been extensively studied over the past ten years, thanks to growing accessibility of molecular biology techniques and publications on the ACCase target gene and relevant DNA markers for the most commercially significant grass weed species [36], [37]. Mutations at seven ACCase codon positions have been shown to confer resistance to at least one ACCase inhibiting herbicide [13]. The first target site mutation identified in most grass weeds is the I1781L mutation probably due to the high level of resistance it confers and the absence of a fitness cost associated with this mutation [38], [39]. In contrast, mutations at codon position 1999 were the last to be identified and the least frequently encountered with only a handful of grass weed populations identified to date [14], [16], [31], [33], [34]. In a comprehensive survey of 54 ryegrass populations and 384 plants from the UK, a single plant was identified that contained the W1999C mutation [33]. The rarity of the 1999 mutations could imply that they are characterised by a fitness penalty. Indeed, in yeast gene replacement experiments, strains carrying the wild type W1999 allele grew two times faster than the equivalent strain with the mutant C1999 allele [14]. That a fitness cost can be associated with the 1999 ACCase mutations is plausible given that tryptophan is very conserved in nature and that a substitution with either serine or cysteine is a rather rare event [40]. Proper fitness trials using wild and mutant 1999 subpopulations created as part of this study and tested under competition as outlined by Vila-Aiub et al. [41] would shed light on the potential fitness penalty associated with the W1999S mutation in *Lolium multiflorum*. The infrequent occurrence of the W1999S mutations could also be due to the fact that it is partially recessive to herbicides such as sethoxydim and cycloxydim, and sensitive to tepraloxydim and clethodim used in rotational dicotyledonous crops grown in the same field. Thus, plants containing the 1999 ACCase mutations are likely to take longer to be selected in the field in particular in an out-breeder species such as *L. multiflorum*. This contrasts with other mutations that confer broader resistance to ACCase herbicides such as the I1781L, D2078G and C2088R mutations. Analysis of a large number of *Lolium* spp. populations from the UK [33] and Australia [42], and *Alopecurus myosuroides* from Europe [30] showed that the D2078G, I2041N and I1781L mutations were the most prevalent respectively. As populations containing the D2078G mutations have been shown to have a fitness cost [39], herbicide use patterns rather that a fitness penalty appears to be a more important determinant regarding the selection of specific mutations in grass weeds subjected to herbicide pressure.

### Detection of mutations at ACCase codon position 1999

Upon discovery of the W1999C mutation which was found to confer resistance to fenoxaprop-P-ethyl only in an *Avena sterilis* resistant population, an allele specific PCR based assay was developed for the high throughput identification of either the tryptophan or mutant cysteine allele in this species [14]. In addition to intrinsic issues associated with allele specific assays [37], the method developed by Liu et al [14] will not allow detection of other nucleotide changes recently found at ACCase codon position 1999. Subsequently, two more robust and universal dCAPS assays were established taking into account the newly identified leucine allele at codon position 1999 [34]. One of the assays uses the same enzyme *Xcm*1 employed here to detect the wild type TGG triplet characteristic of the tryptophan amino acid residue. The second ‘cysteine’ (TGC/T) assay uses the *Xcm*1 dCAPS primer but with an extension of an additional two bases at the 3′ end of the primer. An unrestricted PCR fragment would indicate the absence of a guanine on the third nucleotide of the triplet or presence of cysteine at ACCase codon position 1999. However these two assays alone will not resolve the ambiguity between leucine (TTG) and serine (TCG) residues. The dCAPS assay developed in this study allows positive identification of the wild type tryptophan and mutant serine alleles. This assay is original in the sense that it uses only one PCR product but two different enzyme reactions to detect the tryptophan and serine amino acid residues at ACCase codon position 1999. This amounts to a significant cost reduction with respect to PCR and further demonstrates the flexibility offered by the dCAPS method [27]. When used in conjunction with the second ‘cysteine’ assay developed by Delye et al [34], the dCAPS method will allow inference and high throughput screening of all possible amino acid changes identified at ACCase codon position 1999 to date.

### Specificity and high levels of resistance conferred by non-target mechanisms

Non target site resistance and in particular metabolism is often believed to confer lower levels of resistance compared to target site resistance [43]. At the field level, this type of resistance mechanism is habitually suspected when the weeds are damaged but grow out of the herbicide treatment. In this study, additional underlying resistance, very likely non-target site based, were detected to diclofop-methyl, potentially due to the accumulation of several minor genes over time. Diclofop-methyl is the least potent of all ACCase herbicides and significant levels of non-target site resistance can be selected in an originally sensitive *Lolium* sp. population in only a few generations [22]. Very high levels of non-target site resistance to diclofop-methyl were also found in a different UK *Lolium multiflorum* population characterised by the D2078G mutation [26]. A clear shift in dose responses was also observed for haloxyfop-P-methyl with target site resistance and non-target site resistance conferring a 3-fold and 45-fold resistance respectively. This is interesting given that haloxyfop-P-methyl is a poorly metabolisable herbicide. Though not significant at practical field rates, non-target site resistance had a bigger impact compared to target site resistance on sethoxydim and tepraloxydim, two other non-selective ACCase herbicides on cereal crops. Additionally non target site resistance is often seen as a threat for current and future herbicides in development since it is increasingly being identified as the most prevalent resistance mechanism and because selection with one herbicide can confer resistance to herbicides belonging to a different mode of action [44], [45]. Here we found that non target site resistance can also be specific even among the cereal selective herbicides. Indeed, non-target site mechanisms conferred resistance to practical field rates of diclofop-methyl and clodinafop-propragyl but not pinoxaden, even though the latter two herbicides use the same safener for crop selectivity.

### Conclusion

This detailed molecular and biological study demonstrates the need for a rigorous methodological approach for identifying and quantifying target site and non-target site resistance in weed populations that have evolved resistance to herbicides. It also underlines the unpredictability of resistance conferred by target site and non-target site mechanisms within and between subclasses of ACCase herbicides. As resistance to ACCase herbicides can be quite specific, rotation between ACCase compounds and ideally with other herbicide modes of action and inclusion of non-chemical weed control approaches will ensure their prolonged sustainability for controlling grass weed species.
